# A call to action: A consensus statement on knowledge gaps and research priorities on the management of women with early onset type 2 diabetes in the preconception, pregnancy and postnatal periods: A report from the Diabetic Medicine Symposium at the Diabetes UK Annual Professional Conference, 2025

**DOI:** 10.1111/dme.70252

**Published:** 2026-03-13

**Authors:** Sara L. White, Danielle Schoenaker, Sharon Mackin, Rita Forde, Angela C. Flynn, Eleanor Dyer, Nicola Heslehurst, Robert Lindsay, Claire L. Meek

**Affiliations:** ^1^ Department of Women and Children's Health, School of Life Course and Population Sciences King's College London London UK; ^2^ Department of Diabetes, Guy's and St Thomas' NHS Foundation Trust UCL Institute for Women's Health London UK; ^3^ School of Human Development and Health, Faculty of Medicine University of Southampton Southampton UK; ^4^ MRC Lifecourse Epidemiology Centre University of Southampton Southampton UK; ^5^ NIHR Southampton Biomedical Research Centre University of Southampton and University Hospital Southampton NHS Foundation Trust Southampton UK; ^6^ School of Cardiovascular and Metabolic Health University of Glasgow Glasgow UK; ^7^ Department of Diabetes Glasgow Royal Infirmary, NHS Greater Glasgow & Clyde Glasgow UK; ^8^ Faculty of Nursing, Midwifery and Palliative Care, King's College London London UK; ^9^ School of Nursing and Midwifery, Brookfield Health Sciences Complex University College Cork Cork Ireland; ^10^ RCSI, School of Population Health Dublin Ireland; ^11^ Population Health Sciences Institute Newcastle University Newcastle upon Tyne UK; ^12^ Leicester Diabetes Centre, Leicester NIHR BRC University Hospitals of Leicester NHS Trust Leicester UK; ^13^ Diabetes Research Centre University of Leicester Leicester UK

**Keywords:** early‐onset type 2 diabetes, neonatal, outcomes, postnatal, preconception, pregnancy

## Abstract

**Aims:**

Research to inform, co‐develop and evaluate optimal care for women with early‐onset type 2 diabetes (EOT2D) before, during and after pregnancy is lacking. Informed by patient perspectives and the results of the James Lind Alliance priority‐setting partnership in diabetes in pregnancy, we aimed to develop a consensus statement to guide future research efforts to meet the needs of women with EOT2D in the preconception, pregnancy and postnatal periods.

**Methods:**

Results from three systematic reviews covering interventional, observational and qualitative studies were presented at the Diabetes UK annual professional conference in Glasgow in February 2025. The results were discussed by an expert panel with audience participation.

**Results:**

There is very limited research to guide care for women with EOT2D, especially in the preconception and postnatal periods. In pregnancy, there have been limited studies assessing interventions, mainly encompassing medication and glucose sensor use, but most are small and have limited generalisability. Observational data suggests that managing glycaemia, addressing maternal BMI and preventing excessive gestational weight gain improve outcomes for women with EOT2D in pregnancy. Qualitative data highlight the negative impact of EOT2D on pregnancy and the need for optimised support. Targeted, innovative and cross‐cultural studies across the reproductive life course are urgently needed to address the short and longer‐term maternal and offspring risks for individuals with EOT2D.

**Conclusions:**

Given the rising prevalence of EOT2D and the risk of adverse pregnancy outcomes for women with EOT2D and their children, prioritising research in the preconception, pregnancy and postnatal periods is vital to ensure that care needs are met to improve health outcomes for women and their children.

**What's new?**

- The prevalence of type 2 diabetes in women of reproductive age is increasing, creating additional support needs for preconception, pregnancy and postnatal care to prevent perinatal complications and improve lifelong health for affected women and their children.
- To prioritise future research efforts, a consensus statement was developed based upon three systematic reviews, which were presented and discussed with an expert panel and audience at the 2025 Diabetes UK conference.
- Several key priorities are needed to urgently improve outcomes, including improving access to preconception care, optimising maternal weight management before, during and after pregnancy and addressing issues of inequity and access to care.

## 1 INTRODUCTION

Type 2 diabetes (T2D) in pregnancy is increasingly common and is associated with an increased risk of adverse events such as miscarriage, stillbirth and neonatal death^1^ as well as other suboptimal pregnancy outcomes including preeclampsia, preterm delivery, instrumental/operative deliveries, small (SGA) and large‐for‐gestational‐age (LGA) and neonatal hypoglycaemia.^2^ In addition, early‐onset T2D (EOT2D; conventionally defined as a diagnosis before 40 years and therefore particularly relevant to reproductive health) carries a high risk of diabetes complications,^3^, ^4^, ^5^ premature cardiovascular disease (CVD),^6^, ^7^, ^8^ multimorbidity^9^, ^10^, ^11^ and mortality,^5^, ^12^, ^13^, ^14^ increasing the medical complexity of pregnancy. However, despite the rising prevalence of EOT2D in the antenatal population,^1^, ^15^ and the clinical challenges of addressing complex medical needs, there remain important evidence gaps about optimal care for this group during the preconception, pregnancy and postnatal periods.

We undertook a series of systematic reviews, summarising the available evidence from interventional, observational and qualitative studies^2^, ^16^, ^17^ and identified key research gaps that should be addressed to improve outcomes in EOT2D pregnancy. These data were presented at the Diabetes UK annual professional conference in Glasgow in February 2025. During the session, we highlighted evidence gaps surrounding preconception, pregnancy and postnatal care and shared epidemiological information from Scotland, showing ongoing rising disease prevalence (Mackin et al., unpublished data). Key points were discussed with the audience during the Diabetic Medicine symposium to generate a consensus statement to support and prioritise future research endeavours.

## 2 UNDERSTANDING THE CONTEXT OF EOT2D IN PREGNANCY

### 2.1 Prevalence in the UK

T2D accounts for over half of the diabetes pregnancies among those with pre‐existing diabetes in England and Wales, affecting around 3000 pregnant women per year. Data collected from England and Wales are published in the largest dataset of diabetes in pregnancy globally.^1^ There are no published data from Northern Ireland at present. In Scotland, 1 in 338 pregnancies in 2021/22 were to mothers with T2D (Mackin et al., unpublished data). Of those, half received their T2D diagnosis under the age of 30 years, whilst almost 1 in 10 were diagnosed under age 20 years (Mackin et al., unpublished data). However, these figures may be an underestimate since most women are not routinely tested for diabetes in early pregnancy, and some women with EOT2D may be misdiagnosed as having gestational diabetes in the third trimester. Women who have a miscarriage may not receive any diabetes testing during pregnancy. Recent work in Scotland has suggested that the true prevalence of EOT2D universally may be much higher. A study evaluating the impact of offering HbA1c and random blood glucose testing in early pregnancy to women who have risk factors for gestational or T2D showed that 3% demonstrate glycaemic parameters compatible with a diagnosis of T2D.^18^ To address this, the Scottish Intercollegiate Guideline Network (SIGN) recently recommended an early pregnancy HbA1c for all women with risk factors for T2D in Scotland to support early diagnosis and intervention, although it stops short of universal testing for all pregnancies.^19^ Improving outcomes in other UK regions will be difficult without more consistent approaches to diagnosis in early pregnancy.

### 2.2 Perinatal mortality and outcomes of EOT2D in pregnancy

Women with EOT2D have suboptimal pregnancy outcomes; data from Scotland in the whole obstetric population with T2D revealed one‐third of women delivering LGA infants, as well as a 20% admission rate to neonatal intensive care units, often for longer than 48 hours, increasing healthcare costs. For those with EOT2D, they were more likely to have an LGA infant (37.8% vs. 31.5% for those diagnosed with T2D under 30 years and ≥ 30 years, respectively), and were more likely to be documented as a smoker in pregnancy (19.2% vs. 13.4%). However, even more concerningly, data from England and Wales demonstrate that the perinatal mortality risk is markedly elevated amongst those with EOT2D in pregnancy. National Pregnancy in Diabetes (NPID) audit data show that of those with the highest HbA1c concentrations in the first trimester (>9%; 75 mmol/mol), around 10% of women will experience a perinatal death (defined as stillbirth or neonatal death; miscarriages not included) and 8% will have a baby with a congenital anomaly.^1^ Rates of perinatal death are much higher in women with EOT2D compared to women with T1D with similar levels of glycaemia, suggesting that tackling hyperglycaemia is only part of the solution. Data from MBRRACE‐UK reports suggest that other factors, such as socioeconomic deprivation, language/education barriers and ethnicity, all contribute to perinatal mortality in this population. Addressing the underlying intersecting inequities in care and outcomes would require structural and social change. Possible solutions might include providing more equitable, flexible access to care, reducing bias and racism within healthcare, improving healthcare professional communication across language/cultural/education barriers and giving extra consultation time to explain more clearly how the system works, and when and how to call for help.

### 2.3 Medical complexity and EOT2D

The increasing prevalence of EOT2D is important because age is a key predictive factor in determining the long‐term effects of T2D on premature mortality and CVD morbidity.^8^, ^13^, ^20^, ^21^ For example, previous work with 23 million person‐years of follow‐up, which included UK Biobank data, found that a diagnosis of T2D at ages 30, 40 or 50 years was associated with 14, 9 and 5 years of life lost in men, and 16, 11 and 7 years of life lost in women, respectively.^22^ While overt CVD in pregnant women with EOT2D is fortunately rare, women commonly have comorbidities such as obesity, hypertension, retinopathy, nephropathy, metabolic‐associated fatty liver disease or PCOS,^3^, ^4^, ^5^ which can lead to more complications in pregnancy, increasing risks for the mother and baby.^1^ Greater medical complexity necessitates greater medical oversight during pregnancy and birth. An audit of primary care reported multiple comorbidities for women with EOT2D: despite multiple exposures to medications not recommended during pregnancy, the provision of contraception was suboptimal.^17^, ^23^

### 2.4 Experiences of care

Women with EOT2D report that their overall pregnancy experiences were negatively impacted by their diabetes.^17^, ^24^ Many indicated that they lacked understanding about the management of their diabetes in the context of care prior to and during pregnancy. They attributed enhanced worry, anxiety, a lack of self‐efficacy, over‐medicalisation of pregnancy, all of which disrupted a ‘normal’ experience of antenatal care, and negatively impacted their self‐management. There was a general sense that the support offered by family members, partners and healthcare professionals was often not tailored to the needs of women with EOT2D,^17^ and they reported feeling stigmatised and judged for having T2D during their reproductive years.^25^ Since around 10% receive the diagnosis for the first time during pregnancy, raising awareness of the risks of EOT2D in pregnancy, the importance of access to preconception and interpregnancy care and setting realistic expectations of pregnancy care all remain key challenges.^25^

## 3 PRECONCEPTION CARE

### 3.1 Existing evidence

While many pregnancies are unplanned, planning and preparing for pregnancy is considered to be a vital step in improving outcomes for women with EOT2D who want to have a baby. However, uptake of preconception care is relatively low in women with EOT2D^1^ primarily due to a lack of awareness of the role of preconception care or services among both individuals and healthcare providers, leading to suboptimal access.^25^ In parallel, our previous work showed that women felt anxious about the effect of EOT2D upon fertility, but lacked understanding of the outcomes of EOT2D in pregnancy, which influenced their views on the value of contraception.^17^ Women received advice on contraception from family and friends, who were well‐meaning but had the potential to pass on misinformation or misunderstandings inappropriate for women with diabetes.^17^ Barriers to preconception care and contraceptive use include access, language, education, socioeconomic status, religious beliefs and cultural practices, which are likely to be particularly relevant in this population.^17^, ^26^ These factors need to be considered when developing effective interventions to support women prevent, plan and prepare for pregnancy, if and when this is relevant to them.

There is limited high‐quality evidence for effective preconception care strategies to support women with EOT2D. Indeed, our systematic review of intervention studies failed to find a single randomised controlled trial set in the preconception period in women with T2D, dating back 25 years.^16^ Our systematic review of observational studies, however, identified that receiving preconception care was associated with higher odds of taking folic acid, higher vitamin D concentrations and reduced gestational weight gain.^2^ Observational evidence also suggests that preconception care might be associated with reduced HbA1c in the first trimester,^2^ but did not influence outcomes such as hypertensive disorders of pregnancy, miscarriage rates or birth outcomes, including delivery modality or timing and infant birthweight.

### 3.2 Key knowledge gaps and priority areas for future research

Raising awareness of preconception care remains a key priority. Robust methods to implement effective and culturally appropriate preconception support across community, primary and secondary care are crucial and need to be achieved at scale. Beyond the challenges of accessing services, for those who successfully attend the preconception clinic, evidence‐based interventions are lacking. Traditional glucocentric approaches favour optimising diet and physical activity, and where necessary, adding insulin to improve glycaemia in preparation for pregnancy. However, insulin use may promote weight gain and there is often a reluctance to inject insulin during the potentially lengthy preconception period, with a preference for oral medications. An alternative weight‐centric approach using GLP‐1 receptor agonists to promote weight loss and improve glycaemia in preparation for pregnancy is increasingly used, with minimal evidence base, and contrary to national guidance. Key questions remain about the safety of GLP‐1 medication use before, during and after pregnancy, the potential for a rebound effect on gestational weight gain if stopping pre‐pregnancy,^27^ and how to support ongoing metabolic health. Strategies for achieving remission of T2D would be transformative in the preconception period, especially for women with recent EOT2D, but this has not been studied. The NHS Path to Remission is likely to be effective but may require optimisation when starting in the preconception (or postnatal) period, as not all women with EOT2D are currently eligible and women may not be willing to delay pregnancy to complete the programme.^28^ It is also increasingly recognised that diabetes might impair male reproductive health outcomes, sperm quality and fertility,^29^ but further research is needed to explore the impact of paternal/couple diabetes on pregnancy and offspring outcomes. Longterm change will require strategic decisions from funders and pharmaceutical companies to increase relevant studies undertaken in this space.

## 4 PREGNANCY CARE

### 4.1 Existing evidence

In pregnancy, women with EOT2D are typically advised to conduct self‐monitoring of blood glucose levels with a glucose meter, receive additional advice on diet and exercise, and undergo treatment with metformin and/or insulin to optimise blood glucose management.^4^ A uniform approach to the use of metformin and insulin in EOT2D management in pregnancy has resulted in a lack of innovative studies evaluating optimal dosing or alternative regimens or alternative medications that may improve glycaemia. Trials of metformin use in EOT2D or women with overt diabetes in pregnancy have shown no clear benefit on neonatal outcomes, but improved secondary outcomes such as improved maternal glycaemia reduced gestational weight gain and caesarean section rates.^30^ Despite metformin use, around 85% of women with EOT2D require insulin during pregnancy.^31^ One trial has shown the benefits of insulin detemir over NPH (neutral protamine Hagedorn) insulin upon neonatal outcomes, hypertensive disorders and maternal hypoglycaemia rates.^32^

Most studies evaluating the efficacy of behaviour change interventions in pregnancy have focussed on gestational diabetes,^33^, ^34^, ^35^ with extrapolation to EOT2D; indeed, evidence about optimising diet, increasing physical activity, improving sleep and reducing sedentary time is currently lacking in women with EOT2D in pregnancy. A single small study suggested that aerobic physical activity for 10 weeks could improve placental blood flow and reduce fasting hyperglycaemia.^36^ Although our systematic review of observational studies identified gestational weight gain as a key modifiable risk factor for suboptimal pregnancy outcomes, there is no current evidence addressing optimal targets for weight gain or specific interventions to promote healthy weight during pregnancy in EOT2D.

While diabetes technology has revolutionised care for women with type 1 diabetes (T1D) during pregnancy, there is limited evidence for its use in EOT2D. Published studies include only small numbers of women with EOT2D, used now‐outdated glucose sensors or used current sensors for only short periods. Larger trials are underway to establish the role of continuous glucose monitoring (CGM) in EOT2D and to set appropriate targets for CGM metrics in this population (clinicaltrials.gov).

### 4.2 Key knowledge gaps and priority areas for future research

The most important clinical risk factors for suboptimal outcomes appear to be related to glycaemia, BMI and gestational weight gain.^1^, ^2^ Moreover, ethnicity and socioeconomic status clearly impact risk, with little evidence of how this might be mitigated.

Given the paucity of the current evidence base and a clear need, devising and testing new interventions to support healthy pregnancy outcomes is essential. There is reason to be cautiously optimistic; a targeted single database search of studies (recruiting/not yet recruiting) identified 11 current relevant studies, mainly encompassing technological and pharmacological interventions (from clinicaltrials.gov). However, there is a need for novel and innovative approaches that go beyond glycaemia, particularly in relation to improving reach and outcomes across the geographic, ethnic and socioeconomic landscape.

## 5 POSTNATAL CARE

### 5.1 Existing evidence

Our three systematic reviews evidenced an essential absence of studies encompassing EOT2D in the postnatal period. A single small intervention study tested the use of tai chi qigong exercises post pregnancy, and a single observational study explored the impact of breastfeeding at 6 months on child weight and BMI, whilst no qualitative studies focusing on the postnatal experience were identified.

### 5.2 Key knowledge gaps

Babies of women with EOT2D have higher rates of neonatal and infant death, but the reasons for this are unclear. It is also unclear if pregnancy or postnatal interventions are likely to be able to improve the outlook for the most severely affected infants. Many studies have explored interventions to prevent the development of EOT2D post‐gestational diabetes, but there is an urgent need for evidence‐based approaches to improve interpregnancy and long‐term health in individuals with EOT2D, as well as their babies.

There are several key priorities in the postnatal period, which require integrated communication between primary and secondary care. Breastfeeding should be encouraged and supported, particularly in women from population groups who are traditionally less likely to breastfeed, such as women from deprived communities. Diabetes medication regimens need to be adjusted for postnatal life, ideally coming off insulin where possible, with a further medication review after breastfeeding has stopped. Future risk of CVD should be assessed, and where appropriate, medications to optimise CVD risk factors such as hypertension, microalbuminuria and hyperlipidaemia should be started or restarted. Commencing contraception is a priority. The postnatal period also provides time to address overweight and obesity by supporting women with diet and physical activity, as well as the use of weight optimising medications such as GLP‐1 receptor agonists. While there is limited data on these medications postnatally, there is increasing evidence that they support successful long‐term weight management in people with diabetes. Navigating planning a subsequent pregnancy in the context of such medication is also under‐investigated. Previous qualitative work has highlighted that women often struggle to navigate health service structures post‐pregnancy when moving from a secondary care specialist environment back to primary care, and this is often a barrier to access.

### 5.3 Priority areas for future research to improve care and outcomes

Women with EOT2D need a coordinated pathway postnatally to ensure their health needs are addressed, coupled with effective long‐term follow‐up, whether planning a further pregnancy or not. Further research exploring postnatal interventions to address weight, cardiovascular risk and to promote the appropriate use of contraception to improve short‐ and long‐term health and prepare appropriately for a subsequent pregnancy is needed. Although there is limited evidence about breastfeeding in women with EOT2D, this is likely to be beneficial, and interventions to promote and prolong breastfeeding in this population would be welcomed.

Optimal care in pregnancy includes access to a specialist dietitian, alongside a diabetologist, obstetrician, midwife and full multidisciplinary team in secondary *and* primary care. Work in other contexts, for example gestational diabetes, has highlighted that women benefit from high levels of healthcare professional support during pregnancy, but often feel abandoned postnatally.^37^, ^38^, ^39^ Research that aims to maintain good levels of support for health goals and glycaemia in women postnatally is needed in order to design systems to care for people with EOT2D who often have complex medical and social needs. Moreover, the postnatal period might prove to be a useful teaching moment to introduce targeted interventions to improve the health of the entire family.

## 6 ADDRESSING SOCIOECONOMIC AND ETHNIC INEQUALITIES TO IMPROVE OUTCOMES IN WOMEN WITH EOT2D

### 6.1 Existing evidence

Women with EOT2D often come from ethnic minority groups, experience cultural and language barriers, and face intersectional inequalities, for example, relating to socioeconomic or educational disadvantages.^25^, ^40^ There is minimal evidence about culturally‐tailored interventions to improve care during the preconception, pregnancy and postnatal periods.^2^, ^16^

### 6.2 Key knowledge gaps

There are evidence gaps in how racism, bias and health service structures might contribute to inequalities in access and delivery of care, resulting in adverse health outcomes across socioeconomic and ethnic groups. There are cultural differences in women's attitudes towards diabetes, contraception, pregnancy planning, dietary management, weight management and physical activity in pregnancy. Moreover, the effect of social and cultural barriers on women's priorities for their care is unclear. Some evidence suggests that culturally‐tailored communication and interventions could improve women's experiences of pregnancy and the postnatal period.^17^, ^25^

### 6.3 Priority areas for future research to improve care and outcomes

Working with the EOT2D community to achieve a greater awareness of socio‐economic and cultural differences and how these might influence women's access to, and outcomes of, care, and how health professionals and systems are structured to deliver care would facilitate the co‐production of effective interventions to address inequalities in risk across groups. Other potential approaches might include addressing healthcare professional bias or racism, improving communication and through provision of culturally‐appropriate information on pregnancy planning, diet and physical activity and weight management, culminating in equitable access to care. A further aspect of interest is peer support, allowing women to have culturally and socially tailored conversations about diabetes in pregnancy in their communities. Although, to our knowledge, there is a lack of evidence on this approach for women with diabetes, it has shown promise in pregnant women without diabetes (Box 1).^41^

BOX 1 **Consensus statement**

- EOT2D in pregnancy is an under‐researched condition that has a high and rising prevalence in the United Kingdom and elsewhere.
- Women with EOT2D are often at high risk of adverse pregnancy outcomes, including higher rates of maternal and perinatal death, but the reasons for this are unexplored.
- Research into improving outcomes for women with EOT2D in the preconception, pregnancy and postnatal periods is urgently needed.
- Much greater focus is needed on weight management before, during and after pregnancy as an opportunity to improve outcomes for mothers and their babies.
- We urgently need co‐developed interventions to improve coordinated preconception, pregnancy and postnatal care, which can be implemented at scale and which are feasible and acceptable to a population with complex medical and social needs and frequent intersectional barriers to accessing optimal care.
- Postnatal care needs to urgently engage with the real risk of premature morbidity and mortality from CVD and diabetes complications in women with EOT2D.

## 7 CONCLUSIONS

There is a paucity of evidence for the management of women with EOT2D in the preconception, pregnancy and postnatal periods, but a rising disease prevalence, associated with multiple potentially preventable complications in women and children who commonly come from under‐represented groups.^1^, ^2^, ^16^, ^17^ As the prevalence of EOT2D in women rises year upon year, we urgently need to raise awareness among patients, families, healthcare professionals and policy makers about EOT2D in pregnancy, and to advocate for funding for research to improve care. Listening to women's voices, especially from communities where the disease prevalence is highest, provides opportunities to create a new dialogue about EOT2D in pregnancy, improving the short and longer‐term health and well‐being of mothers and children.

## AUTHOR CONTRIBUTIONS

CLM and SW drafted the statement and all authors revised and reviewed the final manuscript.

## FUNDING INFORMATION

CLM is supported by the European Foundation for the Study of Diabetes—Novo Nordisk Foundation Future Leaders' Award (NNF19SA058974). CLM's work on (T2D) in pregnancy is supported by Diabetes UK under project grant number 24/0006695. S.L.W. is supported by a grant from the Medical Research Council (UK) (MR/W003740/1). DS is supported by the National Institute for Health and Care Research (NIHR) through an NIHR Advanced Fellowship (NIHR302955) and the NIHR Southampton Biomedical Research Centre (NIHR203319). NH is supported by the NIHR Academy through a Career Development Fellowship (CDF‐2019‐11‐ST2‐0011).

## CONFLICT OF INTEREST STATEMENT

There are no conflicts to declare.

## Supporting information


Data S1.

## Data Availability

Data are available upon request, subject to approval from study steering groups.
