# Supplementary material for: A call to action: A consensus statement on knowledge gaps and research priorities on the management of women with early onset type 2 diabetes in the preconception, pregnancy and postnatal periods: A report from the Diabetic Medicine Symposium at the Diabetes UK Annual Professional Conference, 2025
Source: Diabet Med. 2026 Mar 13;43(5):e70252. doi: 10.1111/dme.70252 (PMC13074131; doi:10.1111/dme.70252)
Supplement: Supplementary file 1 — Data S1. [file DME-43-e70252-s001.docx]

**Supplementary Information.**

**Title: A call to action: a consensus statement on knowledge gaps and research priorities on the management of women with early onset type 2 diabetes in the preconception, pregnancy and postnatal periods.**

Information about ongoing trials relevant to the prepregnancy, pregnancy and postnatal care of women with early-onset type 2 diabetes (EOT2D).

**INTERVENTIONAL STUDIES / TRIALS**

*Technology*:

**NCT06005987:** Mobile app logging for diabetes in pregnancy: T2 and GDM – RCT – traditional paper logging vs OneTouch Reveal smart phone app – primary outcome compliance – actual vs expected logged glucoses. Secondary – in range over 4 weeks. 40 women – Augusta, Georgia, US. 2024 start – 2027 expected end

NCT05001815: Peking, China. CSII versus MDI in pregnant women with T2DM. Single centre, open label RCT. 80 women. Registered 2021, but apparently not yet recruiting.

*Medication / Treatment*:

**NCT06619301:** RCT Glargine vs NPH for treatment of DM in Pregnancy (GDM or T2) – Started recruitment 2024, planned end 2027, 160 women, open label non inferiority, prospective RCT. Illinois US. Primary outcome – maternal Hypoglycaemia episodes. Secondary – LGA/Shoulder dystocia

**NCT05124457:** Detemir vs NPH (DETERMINE) – Los Angeles US, 336 women – start 2022, due to complete mid 2025. Open label RCT. T2 and GDM needing insulin. Primary outcome – Neonatal hypoglycaemia, prolonged hypoglycaemia

**NCT06319560:** Hydroxychloroquine in T2D during pregnancy – Malaysia – Improvement in glucose control with hydroxychloroquine in type 2 diabetes during pregnancy: a randomised controlled trial – open label – 200mg – primary outcome HbA1c, fructosamine, IL6, IL10, TNF, shearwave elastography of placenta – ‘does HXQ improve pregnancy outcomes, does it improve inflammatory markers’ – 56 women, recruiting –2024 - end 2027

**MIMICH** Metformin impact on maternal and infant cardiometabolic health **ISRCTN13866189**. RCT open label. In women with diabetes in pregnancy and risk factors for placental disease, what is the effect of withholding treatment with metformin vs usual care including metformin on fetal growth and maternal cardiometabolic health during pregnancy? Intervention – diet&lifestyle+/-insulin vs standard care – diet, lifestyle, metformin and insulin. Primary outcome = third trimester fetal growth velocity change in fetal growth zscore between 20 and 26 weeks and birth. 2021 – 2026. 225 women. T2Dm or early GDM – presence of at least 1 risk factor for placental disease – raised BP, abnormal pulse wave velocity etc

**CTRI/2024/03/064907**: To compare two different insulin regimes for better pregnancy outcome – premixed split insulin Mixtard 30% rapid acting, 70% isophane vs MDI – actrapid and insulatard insulin regime – RCT – 50 individuals – India – Hypoglycaemia and glucose control at 28 – 30 weeks and 34 – 36 weeks

**NCT02932475: MOMPOD**: <https://clinicaltrials.gov/study/NCT02932475> - published Dec 2023 - <https://jamanetwork.com/journals/jama/fullarticle/2812641> - 794 pregnant adults with t2 diabetes or diabetes identified in early pregnancy – randomised to metformin added to insulin vs placebo. No reduction in composite neonatal adverse outcome (perinatal death, preterm birth, LGA or SGA, hyperbilirubinaemia), but resulted in fewer LGA infants. 17 US centres. Study halted at 75% accrual for futility in detecting a significant difference in primary outcome.

*Nutrition/Lifestyle:*

**NCT06218147:**  Community based nutrition lifestyle therapy for pregnant Latina women with diabetes. California, US. 2024 – 2025. 30 participants. Latina women – development and testing culturally-tailored dietary lifestyle behavioural intervention that supports eating plant-based foods instead of processed foods, implemented through community health workers to prevent excess GWG and help with glucose control. Randomised. Standard care vs nutrition-behaviour lifestyle program. Primary outcome – percent TIR using CGM worn at 3 time points for 30 days each. Secondary measure – GWG

**NCT05979519**: Fresh carts for Mom’s to improve food security and glucose management: 2023 – completed recruitment – RCT – 22 women – Kentucky US – GDM or T2 – 3 arms – 1) medically tailored frozen meals – 10 frozen meals delivered by door dahs to their home each week for 12 weeks, 2) Fresh Funds for Mom’s – Instacart fresh funds program – can select foods eligible in the fresh funds to be delivered to their home each week for 12 weeks. 3) standard care. Primary outcome – change between fasting blood glucose at baseline and post intervention. Secondary – food insecurity…

**TCTR20190802002**: Efficacy of nutrition therapy for women with gestational diabetes and type 2 diabetes mellitus and pregnancy outcomes. Thailand. Registered 2019, not yet recruiting, but updated as pending earlier this year. 200 women – Non randomised – nutrition and lifestyle + ‘closed monitoring of blood sugar’ – primary outcome fetal macrosomia

*Group care:*

**NCT03301792:** Group versus traditional prenatal care (usual diabetic clinic attended by residents and faculty) for diabetes. (finished recruiting). Missouri, US. RCT – 117 women – group prenatal care vs traditional care on glycaemic control and postpartum weight retention in T2 and GDM. Group visits every 2 weeks on continuous cycle through a six-session curriculum – weekly visits from 37 weeks? 2 – 12 women 2hr visits – pregnancy, behavioural health, diabetes and nutrition education. Educator and obstetric provider. Primary outcome – glycaemia – mean fasting blood glucose, mean 1hr, HbA1c at delivery, 4 – 12 weeks, 6 months and 1 yr postpartum. And postpartum weight retention.

*Continuous glucose monitoring (CGM):*

**ISRCTN12804317: PROTECT** – RCT – pregnancy outcomes using continuous glucose monitoring technology in pregnant women with early onset type 2 diabetes: a multicentre RCT of the clinical and cost-effectiveness of using CGM in pregnant women with early-onset type 2 diabetes. Continuous glucose monitoring amongst pregnant women with early-onset type 2 diabetes – East Anglia – 422 women – CGM vs standard care – 2023 – 2027 - >16, HbA1c >=43. Primary outcomes - TIR, neonatal unit admission of death.

**CGM2: NCT06628453** CGM for management of t2 diabetes in pregnancy. Alabama – Dexcom G7 – 7 US centres – completion 2029 – 564 women. Not yet recruiting.

**NCT05317585: Continuous glucose monitor use in pregnancy –** high risk t2 using insulin – Massachusetts, US, RCT 180 women, CGM vs multiple daily HBM – LGA, maternal glucose control, patient satisfaction, additional perinatal outcomes started 2024 – end 2027 HbA1c >6.5% or OGTT 126 and 200. CGM vs standard care – primary outcome LGA 2024 – 2027. Device not specified.

**NCT05947916:** RtCGM system in T2DM with pregnancy, China. started recruitment 2022, estimated completion 2024 – 240 women. Multicentre, open label trial. Intervention – monitor blood glucose – more than 50% of the time every 4 weeks. Vs SMBG. Medtronic iPro2. Primary outcome – TIR

**NCT05662462: ACHIEVE RCT** Successfully achieving and maintaining euglycaemia during pregnancy for t2 diabetes through technology and coaching – Ohio US. 2024 – 2029 124 women. Intervention – mHealth app with CGM Dexcom G7, provider dashboard and care team coaching) vs standard care – prenatal visits, self-monitored blood glucose, diabetes care and education specialist. Inclusion criteria hba1c >6.5%– primary outcome proportion of individuals who achieve HbA1c <6.5% in 3^rd^ trimester.

**CTRI/2023/06/053949:** Comparison of lifestyle modification with routine care and comparison of benefit of use of technology with usual care. To evaluate the effectiveness of enhanced behavioural intervention and use of technology (Continuous glucose monitoring) for improving pregnancy outcomes in women with type 2 Diabetes mellitus: A three-arm parallel Randomised Controlled Trial .120 women. RCT – video assisted group sessions on diet and exercise 2) CGM 3) usual care. Primary outcome difference in HbA1c from randomisation to 34 – 38 weeks**. 2023, India**

**CTRI/2021/05/03355:** A Study to compare glycaemic control achieved by Flash Glucose Monitoring and Self-Monitoring of blood Glucose in pregnant women with Diabetes Mellitus. India. Target 70. Registered 2021, no evidence of recruitment

*Corticosteroids:*

**NCT04542148:** Glycaemic control after antenatal corticosteroids in women with pregestational and gestational diabetes. 4 sites US. 2022 – est 2025. 120 women. Bridging gap of understanding maternal and neonatal effects of antenatal steroids in women with threatened preterm birth who have diabetes. Women hospitalised for ANS. 3 arm – sliding scale insulin (on top of regular insulin), (plus masked CGM), titration of home insulin, or continuous insulin infusion (off regular insulin). Primary outcome - TIR

*Birth:*

**NCT03912363: US study** Open label RCT Intrapartum glycaemic control with insulin infusion versus rotating fluids – different protocols for IV fluids due to finish March 2025 started recruiting 2019– enrolled 114 women – impact on neonatal glycaemia from birth to 2hrs of life – GDM and T2

*Glucose targets:*

**NCT04672031: Glycaemic targets for pregnant women with GDM and T2DM,** Southern Carolina, RCT ADA/ACOG targets (fasting =<95mg/dl, 1hr =<140mg/dl ) 5.3/7.8 versus more aggressive care with lower glycaemic targets (fasting -<80mg/dl and 1hr =<110mg/dl) 4.4/6.1 Primary outcome 250g difference in birthweight between two study arms. 120. Started recruitment 2021, should have finished 2023 – no data available

**COHORT STUDIES**

**NCT06147466**: Time spend in the target range and maternal and neonatal effects in women with type 2 diabetes in pregnancy (TIMELY) – Canada (Feig) – end 2026 – prospective cohort – 50 women – Dexcom – To determine TIR compared to T1 targets – maternal and offspring outcomes

**ACTRN12622001159741**: Evaluation of SFlt/PLGF ratio in women with pre-existing diabetes to identify those at risk of placental insufficiency in order to optimise their antenatal care. DIPPS Diabetes in pregnancy: routine screening for placental insufficiency using the sFlt-1/PlGF ratio. Just finished recruiting – T1 and T2 diabetes. 98 recruited. Blood sampling at 4 weekly intervals from 20 to 36 weeks. Assessment of prevalence of asymptomatic placental insufficiency. Blinded. New Zealand. Looking to see if beneficial to introduce ratio to screen for placental insufficiency in pregnant women with type 1 or type 2 diabetes.

**INSULIN SENSITIVITY STUDIES**

<https://trialsearch.who.int/Trial2.aspx?TrialID=NCT04924504> - Mechanisms Behind Severe Insulin Resistance During Pregnancy in Women With Glucose Metabolic Disorders (SIR-MET)

Pregnancy outcomes and maternal insulin sensitivity (PROMIS) – Groningen, https://clinicaltrials.gov/study/NCT04315545
